# Racial Differences in Serum Adipokine and Insulin Levels in a Matched Osteoarthritis Sample: A Pilot Study

**DOI:** 10.1155/2016/8746268

**Published:** 2016-05-03

**Authors:** Rajiv Gandhi, Anirudh Sharma, Mohit Kapoor, Kala Sundararajan, Anthony V. Perruccio

**Affiliations:** Arthritis Program, University Health Network and University of Toronto, Toronto, ON, Canada M5T 2S8

## Abstract

*Background*. In an attempt to correlate biomarkers with disease, serum-based biomarkers often are compared between individuals with osteoarthritis (OA) and control subjects. However, variable results have been reported. Some studies have suggested an association between certain adipokines and insulin and OA. We know that there are racial differences in OA prevalence and incidence, and from general population-based studies, those of Asian race consistently demonstrate a unique adipokine/insulin serum concentration profile as compared to Caucasians. Whether similar racial differences exist within OA samples is unknown and may have implications for selecting appropriate controls in comparative studies.* Methods*. Serum levels of adipokines, leptin, and adiponectin, along with insulin, were determined by ELISA in patients scheduled for total hip or knee replacement surgery for OA. Fifteen Asian patients were matched 1 : 1 on age (±2 years), gender, body mass index (±1.5 kg/m^2^), and surgical joint with Caucasian patients. Differences in serum concentrations were tested using paired *t*-tests.* Results*. Serum leptin and insulin levels were significantly higher in Asians compared to Caucasians (*p* < 0.05). While serum adiponectin levels were lower among Asians, the difference did not reach statistical significance (*p* = 0.12).* Conclusion*. Findings from this work suggest that when studying serum biomarker concentrations in OA versus controls, race may be an important factor to consider. Our findings warrant confirmation in larger studies.

## 1. Introduction

Serum-based biomarkers often are studied to understand normal biologic processes, pathogenic processes, or pharmacologic responses to a therapeutic intervention [1]. To identify markers specific to certain diseases, disease processes, or disease symptoms, first steps usually entail a comparison between the disease group of interest and a healthy control sample. Such a method has been used in several studies assessing potential associations between serum biomarker concentrations of leptin, adiponectin, and insulin in osteoarthritis (OA) [2, 3]. Findings have been inconsistent, however, with some suggesting likely associations and others suggesting no association when comparing OA subjects with controls for disease presence and/or progression [2–5].

Consistently, general population-based studies have found increased serum concentrations of leptin and insulin levels among those reporting being of Asian race compared to Caucasian race, adjusting for age, gender, and body mass index (BMI) [6]. Furthermore, the prevalence of insulin resistance and type 2 diabetes is greater among Asians compared to Caucasians, even when controlling for body weight [7, 8]. Similarly for OA, racial differences in prevalence and severity have been reported [9–12]. While the understanding associating diabetes with OA is growing [13], leptin and adiponectin have both been well linked to OA disease and symptoms [14–17].

In this pilot study we investigated serum concentrations of the adipokines, leptin, and adiponectin (among the most often studied adipokines in OA [18]) and insulin (insulin resistance related factor) in a late stage hip/knee OA sample comparing Asians and Caucasians, with a view to assessing whether similar differences as found in general population samples exist.

## 2. Methods

This is a secondary analysis of data collected from patients scheduled for hip or knee arthroplasty surgery and consecutively recruited for a prior study [14]. In brief, patients were eligible for the study if they were aged 18 years or older and surgery was for primary OA. All patients had Grade III or Grade IV OA as defined by the Kellgren-Lawrence radiographic rating scale [19]. Patients undergoing surgery for a diagnosis other than primary OA or unable to give informed consent were excluded. Informed consent was obtained from eligible subjects by an independent study coordinator not involved in the patient's medical care. The local hospital Institutional Review Board approved the study protocol.

### 2.1. Questionnaire

All subjects completed a self-report questionnaire at most 6 weeks prior to surgery which elicited demographic characteristics, including age, gender, and race, as well as weight and height, from which BMI (kg/m^2^) was calculated. Participants were asked to respond yes/no to a list of chronic conditions for which they were currently receiving treatment [20].

Participants were selected for this study if they reported being of Asian race. Asian race was determined by a positive response by the patients as to whether they were “South Asian (e.g., Indian, Pakistani, and Bangladeshi), East Asian (e.g., Chinese, Korean, and Taiwanese), or Southeast Asian (e.g., Filipino, Indonesian, Thai, and Vietnamese).”

We then randomly selected participants from among those reporting being “Caucasian/White” matched 1 : 1 on age (within 2 years), BMI (within 1.5 kg/m^2^), gender, and surgical joint to the Asian participants.

Knee-specific pain and function were assessed using the Western Ontario McMaster University Osteoarthritis Index (WOMAC) pain and function subscales, respectively. The WOMAC has high validity and responsiveness for use in lower-extremity OA populations [21, 22]. Subscale scores range from 0 to 100, with higher scores indicating greater limitations/pain.

### 2.2. Serum Collection

Fasting blood was obtained from all participants prior to surgery, stored at −80°C, and analyzed in batch. Plasma concentrations of biomarkers were quantitated by ELISA (human leptin and insulin multiplex ELISA Adipokine Panel 2, Bio-Rad, and adiponectin, multiplex ELISA Adipokine Panel 1, Bio-Rad, USA). Samples were diluted as appropriate and assayed in duplicate in the same run. Serum leptin and adiponectin were selected for comparison in this study as they are the most commonly studied adipokines in OA presently [18].

### 2.3. Statistical Analysis

Descriptive statistics were generated. Continuous variables (age, BMI, adipokine, and insulin levels) were compared between racial groups using paired Wilcoxon signed-rank test. Categorical variables (gender and prevalence of diabetes) were compared with McNemar's test for paired samples. All statistical analyses were performed with SAS (version 9.2).

## 3. Results

Thirty participants were included in the analysis, 15 Asians and 15 Caucasians. The description of the sample is provided in Table 1, along with results from difference testing between racial groups. No differences were found for matching variables (Table 1). Pain and functional limitation were not clinically or statistically different between groups (Table 1).

**Figure 1:**
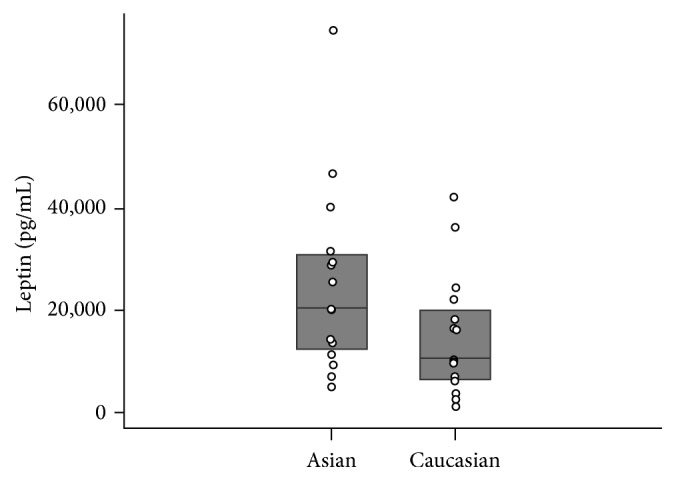
Serum leptin levels compared across races.

**Figure 2:**
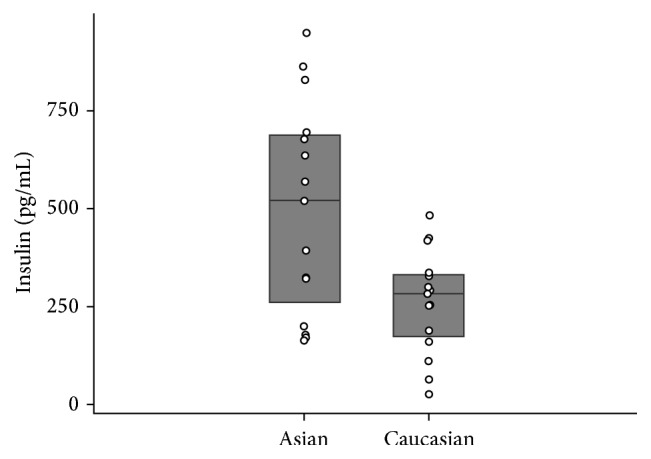
Serum insulin levels compared across races.

**Figure 3:**
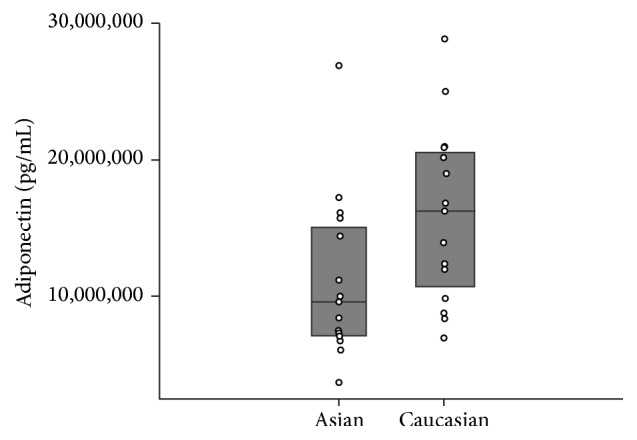
Serum adiponectin levels compared across races.

Serum leptin and insulin concentrations were significantly higher among Asian participants compared to Caucasians (*p* = 0.044 and *p* = 0.034, Figures 1 and 2, resp.). On the other hand, adiponectin concentration was lower among Asians compared to Caucasians, though this difference was marginally nonsignificant (*p* = 0.12) (Table 1, Figure 3).

## 4. Discussion

Serum biomarker differences are consistently sought between OA and control subjects to identify any that may be informative to improving our understanding of OA disease and/or symptoms. Known differences exist in OA prevalence and reported pain levels across race [9–12, 23]. Our findings in an end-stage OA population mirror those found in general population cohorts [6] whereby Asian participants have significantly greater serum leptin and insulin levels than Caucasians. Overall differences in serum concentrations of specific markers between races can have implications for the selection of controls in studies evaluating potential biomarkers of disease and particularly for diseases which vary with race, as is the case in OA.

Insulin resistance (characterized by higher serum insulin levels) is strongly linked to abdominal and visceral obesity [3], and adipose tissue is known to release adipokines, including leptin and adiponectin, into the systemic circulation. Both of these adipokines have been linked to OA disease and symptoms [14–17]. Potential explanations for our findings of greater serum leptin and insulin levels and lower serum adiponectin in Asians compared to Caucasians include variance in adipocyte structure (hyperplastic versus hypertrophic cells) and/or body composition and distribution of adipose tissue between groups [24]. Variations in diet across races may also impact serum insulin and adipokine levels; high glycemic index carbohydrate foods provide the majority of energy in Asian diets (South and East Asian) [24, 25]. Consumption of high glycemic index foods can be associated with low adiponectin levels and high serum insulin and leptin levels [26, 27].

Cognizant of differences in disease profiles by race, together with the knowledge that specific serum-based markers vary by race [9–12, 23], this suggests that race likely is a critical factor to consider when selecting control subjects in comparative OA studies. Conflicting evidence exists, for instance, for an association between serum adipokines and hand OA. One group has suggested that lower serum adiponectin levels, but not leptin, are associated with progression of hand OA [28], while others have suggested no relationship [29] or even that a greater serum adiponectin level is associated with a greater prevalence of erosive hand OA [30]. Our findings suggest a potential need to consider the effects of race; however confirmation of findings in larger samples is warranted.

Insulin and insulin-like growth factor 1 (IGF-1) share structure and functional homology; both are growth promoting proteins [31]. Both have been suggested to have a role in OA pathogenesis [31]. Furthermore, insulin is known to directly increase serum leptin production [32]. Conflicting evidence exists for an association between serum IGF-1 levels and prevalent radiographic knee OA, however [3, 5, 33]. These studies did not consider potential differential effects as they relate to race.

Though our sample was limited in size, we nonetheless found statistically significant differences across Asians and Caucasians. The sample was limited to individuals with end-stage disease as well. Whether findings are generalizable across degrees of OA severity is unknown and warrants further investigation, as do comparisons with healthy controls. Future investigation with greater participant numbers will also facilitate distinctions among Asians from different geographic regions (e.g., East Asian and South Asian). Finally, the study was cross-sectional in nature and addresses association only.

In conclusion, within this matched end-stage knee/hip OA pilot study, Asians had higher serum leptin and insulin concentration compared to Caucasians. Given suggested associations between these markers and OA, comparative OA versus non-OA studies may need to consider race when selecting appropriate non-OA controls. Our findings suggest that a larger study may be warranted to explore racial differences further.

## Figures and Tables

**Table 1:** Description of sample and serum biomarker concentrations.

	Caucasian *n* = 15	Asian *n* = 15	*p* value
Mean (SD) age in years	62.5 (8.9) (range 50–77)	62.1 (9.6) (range 48–78)	*p* = 0.5
Gender: *n* (%) male	7 (46.7%)	7 (46.7%)	*p* = 1.00
Surgical joint: *n* (%) knee	12 (80.0%)	12 (80.0%)	*p* = 1.00
Mean (SD) BMI kg/m^2^	29.8 (4.2) (range 24–41)	29.8 (4.4) (range 25–40)	*p* = 0.95
Diabetes *n* (%)	2 (13.3%)	3 (20%)	*p* = 1.00
Mean comorbidity count (SD)	2.2 (1.5) (range 0–5)	2.3 (1.5) (range 0–5)	*p* = 1.00
Median (range) serum leptin (pg/mL)	10,264.9 (1,221.12–36,181.4)	20,208.2 (5030.6–46,543.5)	*p* = 0.044
Median (range) serum adiponectin (pg/mL)	16,264,000 (6,946,204–82,845,000)	9,391,300 (3,714,488–26,940,000)	*p* = 0.12
Median (range) serum insulin (pg/mL)	284.8 (27.1–483.9)	520.9 (164.5–949.2)	*p* = 0.034
Mean WOMAC knee pain (SD)	52.0 (21.4)	56.8 (16.8)	*p* = 0.54
Mean WOMAC knee function (SD)	47.5 (22.4)	49.8 (18.9)	*p* = 0.77
